# BMP-7 Attenuates Inflammation-Induced Pyroptosis and Improves Cardiac Repair in Diabetic Cardiomyopathy

**DOI:** 10.3390/cells10102640

**Published:** 2021-10-02

**Authors:** Ibrahim Elmadbouh, Dinender K. Singla

**Affiliations:** Division of Metabolic and Cardiovascular Sciences, Burnett School of Biomedical Sciences, College of Medicine, University of Central Florida, Orlando, FL 32827, USA; Ibrahim.Elmadbouh@ucf.edu

**Keywords:** BMP-7, pyroptosis, diabetic cardiomyopathy, heart function, cytokines

## Abstract

In the present study, we investigated a novel signaling target in diabetic cardiomyopathy where inflammation induces caspase-1-dependent cell death, pyroptosis, involving Nek7-GBP5 activators to activate the NLRP3 inflammasome, destabilizes cardiac structure and neovascularization. Furthermore, we explored the therapeutic ability of bone morphogenetic protein-7 (BMP-7) to attenuate these adverse effects. C57BL/6J mice (*n* = 16 mice/group) were divided into: control (200 mg/kg, 0.9% saline intraperitoneal injection, i.p.); Streptozotocin (STZ) and STZ-BMP-7 groups (STZ, 200 mg/kg, i.p. injection). After 6 weeks, heart function was examined with echocardiography, and mice were sacrificed. Immunostaining, Western blotting, H&E, and Masson’s trichrome staining was performed on heart tissues. STZ-induced diabetic cardiomyopathy significantly increased inflammasome formation (TLR4, NLRP3, Nek7, and GBP5), pyroptosis markers (caspase-1, IL-1β, and IL-18), inflammatory cytokines (IL-6 and TNF-α), MMP9, and infiltration of monocytes (CD14), macrophage (iNOS), and dendritic cells (CD11b and CD11c) (*p* < 0.05). Moreover, a significant endothelial progenitor cells (EPCs) dysfunction (c-Kit/FLk-1, CD31), adverse cardiac remodeling, and reduction in left ventricular (LV) heart function were observed in STZ versus control (*p* < 0.05). Treatment with BMP-7 significantly reduced inflammasome formation, pyroptosis, and inflammatory cytokines and infiltrated inflammatory cells. In addition, BMP-7 treatment enhanced EPC markers and neovascularization and subsequently improved cardiac remodeling in a diabetic heart. Moreover, a significant improvement in LV heart function was achieved after BMP-7 administration relative to diabetic mice (*p* < 0.05). In conclusion, BMP-7 attenuated inflammation-induced pyroptosis, adverse cardiac remodeling, and improved heart function via the TLR4-NLRP3 inflammasome complex activated by novel signaling Nek7/GBP5. Our BMP-7 pre-clinical studies of mice could have significant potential as a future therapy for diabetic patients.

## 1. Introduction

The prevalence of diabetic cardiomyopathy is found to be ~17% of the diabetic population, with a significant increased risk of heart failure in type-1 and type-2 diabetes [1]. The incidence rate of mortality from heart failure, arrhythmia, and sudden death is increased due to diabetic cardiomyopathy in type-2 diabetic patients compared to non-diabetic patients [2,3]. Diabetic cardiomyopathy is characterized by an alteration of cardiac structure and left ventricular dysfunction in the absence of other cardiac risk factors such as coronary artery diseases or hypertension [1]. The progression of diabetic cardiomyopathy occurs through inflammation, oxidative stress, and lipotoxicity that induces structural alterations in a diabetic heart [1,2,3]. However, the exact nature of inflammation in the diabetic heart and its involvement in the disease progression remains unknown.

Apoptosis and necrosis are widely recognized forms of cell death found in cardiomyopathy [4]. However, whether the presence of inflammation involves the recently recognized pyroptotic cell death in the diabetic heart [5,6] has gained attraction, but detailed understanding on inflammatory cells and the pyroptotic pathway remains unknown. The initiation of pyroptosis is characterized by an inflammasome complex formation, caspase-1 and IL-1β found in various diseases [7,8]. The prevalence of the pyroptotic pathway as well as addition signaling molecules in diabetic cardiomyopathy is complex and understudied. Therefore, the cutting-edge research that we present in this paper aims to understand those molecular mechanisms and establishes a potential therapeutic agent to attenuate such a pathway.

Recent heart studies suggest that there is a presence of sterile inflammation involving the upregulation of endogenous inducers called damage-associated molecular patterns (DAMPs) [8,9]. These upregulated DAMPs can be initiated by apoptosis, necrosis, or other cellular processes that occur when organs are challenged by an injury [10]. Subsequent to DAMPs initiation, inducers directly bind to toll-like receptor-4 (TLR4)- nucleotide-binding oligomerization domain-like receptor (NLR) pyrin domain containing 3 (NLRP3) complex and initiate the pyroptotic cascade [8,9,11]. Recently, NIMA-related kinase 7 (Nek7) was found to have a role in oligomerization and activation of NLRP3 inflammasome complex [12,13]. Additionally, guanylate binding protein-5 (GBP5) is an activator for NLRP3-ASC (apoptosis-associated speck-like protein containing a caspase recruitment domain) oligomerization complex [14]. The exact role of Nek7 and GBP5 in the heart as well as in diabetic cardiomyopathy has never been examined as per the best of our knowledge. Therefore, the present study investigates whether diabetic cardiomyopathy involves sterile inflammation and causes the upregulation of NLRP3-Nek7-GBP5 inflammasome complex, which finally initiates caspase-1-dependent pyroptosis in diabetic cardiomyopathy. We also investigate the source of inflammation and whether an increase in inflammation-induced pyroptosis has any adverse effects on diabetic cardiac remodeling, endothelial progenitor cells (EPCs), neovascularization, and cardiac function.

We and others have recently reported that exogenous bone morphogenetic protein-7 (BMP-7; Osteogenic protein-1; OP-1) inhibits inflammation in pre-diabetic cardiomyopathy [4,7]. Moreover, studies on BMP-7 attenuating diabetic nephropathy [15] and atherosclerosis [16] are gaining significant attention in the current literature due to BMP-7 being a commonly used clinical drug to treat patients with osteoporosis and nonunion bone fracture [17]. It is unknown whether BMP-7 treatment can attenuate inflammatory cells, pyroptosis, inflammation, adverse cardiac remodeling, and enhances neovascularization, ultimately improving diabetic cardiomyopathy.

Therefore, we hypothesized that BMP-7 can inhibit the NLRP3 inflammasome complex and their activator Nek7-GBP5, and the subsequent cascade of pyroptosis in diabetic cardiomyopathy. We also hypothesized that BMP-7 will attenuate inflammatory infiltrated dendritic and M1 macrophages, reducing inflammation and adverse cardiac remodeling while improving heart function. Our study further confirms that the mechanisms of improved adverse cardiac remodeling and heart function are mediated through enhanced neovascularization following BMP-7 treatment, which was not tested before. These series of beneficial effects with BMP-7 in diabetic cardiomyopathy could make this osteoporosis drug a future added treatment to diabetes.

## 2. Materials and Methods

### 2.1. STZ-Induced Diabetes Mellitus in Mice

**Figure 1:**
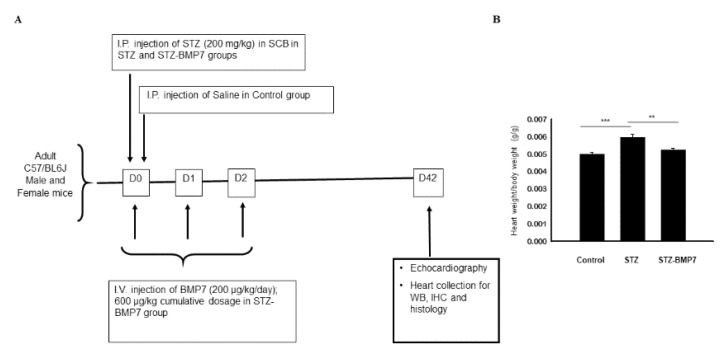
Design of experimental study and BMP-7 reduces heart weight in diabetic heart. (**A**) A schematic image showing the study design, doses, and injection schedule. (**B**) The graph shows the ratio of heart weight-to-body weight (g/g) of all mice after 6 weeks of STZ injection. Statistical analysis was performed using One-Way ANOVA, which was followed by Tukey test; Error bars = mean ± S.E.M. ** *p* < 0.01, *** *p* < 0.001; *n* = 16/group. BMP7: bone morphogenetic protein-7; D: day, IHC: immunohistochemistry; SCB: sodium citrate buffer; STZ: streptozotocin; WB: Western blotting.

All animal procedures conducted in this study were according to a protocol approved by the IACUC (Institutional Animal Care and Use Committee), University of Central Florida, which conforms to the National Institutes of Health (NIH) guidelines. C57BL/6J mice (10 ± 2 weeks old, *n* = 16 animals/ group, equal number of male and female/group) were divided into 3 groups consisting of control, streptozotocin (STZ, diabetic group), and STZ-BMP-7. Control animals were administered with 0.9% saline via intraperitoneal (i.p.) injection. For STZ and STZ-BMP-7 groups, animals were administered with 200 mg/kg STZ (MP Biomedicals, LLC) dissolved in 0.1 M sodium citrate buffer via i.p. injection, whereas STZ- BMP-7 animals were additionally treated with an intravenous (i.v.) injection of 200 µg/kg/day mouse recombinant BMP-7 protein (Bioclone Inc., San Diego, CA, USA) dissolved in phosphate buffer saline, PBS, Gibco) for 3-days (600 µg/kg, cumulative dosage) (Figure 1A), as we published previously [4]. Six weeks (Day-42) following the STZ injection in all animals, echocardiography was performed. Mice were euthanized with 4% isoflurane for 10 min then cervical dislocation was performed. Hearts from each group were harvested, upper part consists of the right ventricle (RV), left ventricle (LV), and atria of the heart stored at −80 °C for Western blots, and the lower part of the heart consists of the RV, and LV were stored at 4% paraformaldehyde at room temperature (RT) for immunohistochemistry.

### 2.2. Immunohistochemistry (IHC) Staining

Hearts fixed with Formalin were washed, processed, embedded in paraffin wax for further histological analysis. Sections (5 µm) were cut and placed on color frost plus slides. Double IHC staining was performed to identify protein marker(s) as previously described [8]. In brief, heart sections were deparaffinized, rehydrated, and blocked for 1 h using a working solution of M.O.M.^®^ IgG Blocking Reagent (1 drop/1.25 mL) following the manufacturer procedures of M.O.M.^®^ Immunodetection Fluorescein Kit (Vector Lab, Burlingame, CA, USA). Then, heart sections were incubated with primary anti-α-sarcomeric actin monoclonal antibody (1:50; Sigma Aldrich, St. Louis, MO, USA city, state abbreviation if USA, country), anti-α smooth muscle (α-SM) actin (costained with CD31) (1:200, Sigma Aldrich, St. Louis, MO, USA ), and c-kit (E3) (1:50, Santa Cruz Biotechnology Inc., Dallas, TX, USA) for 30 min at RT to specifically stain for cardiomyocytes. Next, sections were incubated with secondary biotinylated anti-mouse IgG antibody (4 µL/mL, M.O.M.^®^ kit, Vector Lab, Burlingame, CA, USA) and Fluorescein Avidin DCS (16 µL/mL, green color reaction, M.O.M.^®^ kit, Vector Lab, Burlingame, CA, USA). Following staining for cardiomyocytes, heart sections were blocked with 10% normal goat serum (Vector Lab) for 1 h prior to incubation with primary antibodies TLR4 (1:50, Abcam, Cambridge, MA, USA), NLRP3 (1:50; LS-Bio, Seattle, WA, USA), caspase-1 (1:50; Abcam, Cambridge, MA, USA), interleukin (IL) -1β (IL-1β, 1:50; Abcam, Cambridge, MA, USA), IL-18 (1:50, Abcam, Cambridge, MA, USA ), IL-6 (1:500, Abcam, Cambridge, MA, USA), tumor necrosis factor-α (TNF-α,1:50, Abcam, Cambridge, MA, USA), matrix metalloproteinase-9 (MMP9, 1:100, Abcam, Cambridge, MA, USA), cluster of differentiation (CD) 14 (CD14, 1:100, Abcam, Cambridge, MA, USA), induced nitric oxide synthetase (iNOS, 1:100, Abcam, Cambridge, MA, USA ), CD11b (1:50, Abcam, Cambridge, MA, USA ), CD11c (1:50, Abcam, Cambridge, MA, USA), CD31 (1:200, Abbiotec, Escondido, CA, USA), and fetal liver kinase 1 (FLK-1, 1:200, MyBioSource, San Diego, CA, USA) overnight at 4 °C. Following primary antibody, heart sections were incubated with secondary antibody, Alexa 568 goat anti-rabbit, for 1h at RT. Finally, slides were covered with solution containing DAPI (4’,6-diamidino-2-phenylindole dihydrochloride, Vectashield^®^, Vector Lab, Burlingame, CA, USA), and cover slipped. Images were taken using the Keyence fluorescent microscope. Quantification was calculated in 5 images/section. Total number of positive cells in 5 images was calculated then divided by total DAPI total DAPI nuclei multiplied by 100 [(total cells^+ve^/total DAPI) × 100].

### 2.3. Western Blot Assay

Western blot was performed as we previously described [4]. Heart tissues were sonicated with RIPA lysis buffer, and the supernatant was collected for protein estimation using the Bio-Rad protein Assay (Bio-Rad, Hercules, CA, USA) and Bio-Rad plate reader (iMark^TM^ Microplate Reader, Bio-Rad, Hercules, CA, USA) at 595 nm wavelength [4]. Protein samples (70 μg) were loaded and run on 10% or 15% sodium dodecyl sulfate -polyacrylamide (SDS-PAGE) gel electrophoresis (130 V for 2 h) and electro-transferred onto polyvinylidene difluoride (PVDF, Bio-Rad, Hercules, CA, USA) membranes using transfer buffer and a semi-dry transfer machine (Bio-Rad, Hercules, CA, USA, 16 V for 1 h). PVDF membrane was blocked for 1 h with 1X-Tris buffered saline, Tween 20 (1X-TBS/T, Thermo Fisher Scientific, Waltham, MA, USA) and nonfat dry milk (5%). Membranes were incubated with primary antibodies diluted in blocking buffer (1X-TBS/T and 2.5% nonfat dry milk) for 24 h at 4 °C for TLR4 (1:1000, Abcam, Cambridge, MA, USA), NLRP3 (1:1000, Abcam, Cambridge, MA, USA), Nek7 (1:5000, Abcam, Cambridge, MA, USA), GBP5 (1:1000, Abcam), caspase-1 (1:1000, Abcam, Cambridge, MA, USA), IL-1β (1:500, Abcam, Cambridge, MA, USA), IL-18 (1:1000, Abcam, Cambridge, MA, USA), IL-6 (1:500, Abcam, Cambridge, MA, USA), TNF-α (1:1000, Abcam, Cambridge, MA, USA), MMP9 (1:1000; Abcam, Cambridge, MA, USA), CD14 (1:200, Abcam, Cambridge, MA, USA), and iNOS (1:250, Abcam, Cambridge, MA, USA). In addition, β-actin (1:1000, Cell Signaling, Danvers, MA, USA) served as the loading control. The primary antibody was detected by incubating with goat anti-rabbit horseradish peroxidase (HRP)-conjugated secondary antibody (1:1000, Cell Signaling Technology, Danvers, MA, USA) diluted in 1X-TBS/T and nonfat dry milk (2.5%) for 1h. PVDF membrane was developed with Pierce^TM^ ECL commercially available substrate (Thermo Fisher Scientific, Waltham, MA, USA) for 1–3 min, and X-ray films were developed. Developed Western blots were scanned, band intensities were measured using densitometry, and analysis was performed through ImageJ 1.39o software (NIH, Bethesda, MD, USA).

### 2.4. Hematoxylin and Eosin (H&E) Staining

Heart sections were stained with H&E staining as published [18]. Heart architectures were assessed for the presence of inflammatory cells and hypertrophy of cardiomyocytes in transverse heart sections. Using ImageJ software, the number of inflammatory cells (blue, hematoxylin nuclei) was counted, and for hypertrophy, cardiomyocyte area (mm^2^) was measured by quantifying 5 images/section.

### 2.5. Masson’s Trichrome Staining

Heart sections were stained with Masson’s trichrome, as we previously reported [4]. Stained heart sections were examined using a microscope, collagen deposition was identified as a blue staining area considered as a fibrotic area and quantified as mm^2^. Collagen was quantified in the heart sections [5 image fields] using ImageJ, and the average area was used to assess the interstitial fibrosis. Heart vascular fibrosis was quantified in a total of 5 vessels (vessel fibrosis/total vessel area × 100).

### 2.6. Determination of Heart Function

Transthoracic echocardiography was performed 6-weeks following the last STZ injection. Animals were anesthetized with 2% isoflurane and placed in supine position. Echocardiography was performed using Sonos equipment (HP-Agilent Technologies Inc, Santa Clara, CA, USA) as we reported previously [4,19,20]. M-mode images from at least 3 consecutive cardiac cycles of the left ventricle (LV) were obtained. These images were used to calculate LV internal dimension at diastole (LVIDd), LV internal dimension at systole (LVIDs), and LV end-diastolic and systolic volumes (EDV and ESV, respectively) using our standard protocols [4,19,20]. Indices of LV systolic functions, including fractional shortening (FS%) and ejection fractions (EF%) were calculated using FS = [(LVIDd − LVIDs)/LVIDd] × 100 and EF = [(LVIDd^3^ − LVIDs^3^)/LVIDd^3^] × 100, respectively.

### 2.7. Statistical Analysis

Statistical analysis of all data was performed using One-Way ANOVA followed by the Tukey test as we published [9,19]. Sigma plot software was used to represent the data in graphs, and values were expressed as means ± SEM. *p* values < 0.05 were considered statistically significant.

## 3. Results

### 3.1. Effect of BMP-7 on Heart Weight in Diabetic Cardiomyopathy

To determine the effect of BMP-7 treatment on cardiomegaly in STZ-induced diabetic mice, the heart weight/body weight ratio was calculated (Figure 1B). Our data showed a significant increase in heart weight/body weight ratio in STZ-induced cardiomyopathy group compared to the control (*p* < 0.001). Moreover, BMP-7 reduced the heart weight/body weight ratio significantly (*p* = 0.001) in diabetic mice suggesting that BMP7 provides beneficial protective effects against STZ-induced diabetic hypertrophy and dilation in the heart (Figure 1B).

### 3.2. BMP-7 Inhibits TLR4-NLRP3 Inflammasome Formation in Diabetic Hearts

**Figure 2:**
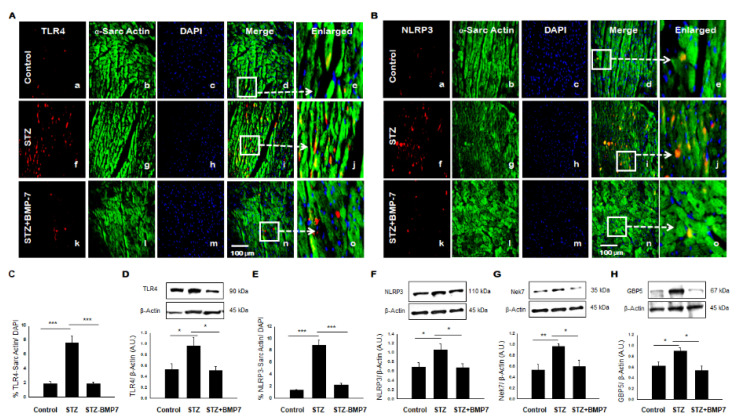
BMP-7 attenuates the expression of TLR4, NLRP3, and NLRP3 activators (Nek7 and GBP5) in the diabetic heart. (**A**,**B**) Representative photomicrographs of heart sections stained in the control group (a–e), STZ group (f–j) and STZ-BMP7 group (k–o) with sarcomeric-α actin (green, stained cardiac myocyte) (b, g, l) and pyroptotic markers TLR4 (**A**) and NLRP3 (**B**) in red (a, f, k) with nuclei stained (c, h, m) by DAPI in blue, merged (d, i, n) and enlarged (e, j, o) image. (**C**,**E**) Quantitative analysis-derived histograms of pryoptotic TLR4 (**C**) and NLRP3 (**E**) +ve cardiomyocytes (colocalization of both red and blue DAPI in green cardiomyocyte) were quantified over total DAPI in heart sections of mice. (**D**,**F**,**G**,**H**) Representative Western blots and densitometric analysis of inflammasome TLR4 (**D**), NLRP3 (**F**), Nek7 (**G**), and GBP5 (**H**) markers are shown. Quantities are presented as an arbitrary unit (A.U). Statistical analysis was performed using One-Way ANOVA, which was followed by the Tukey test. Error bars = mean ± S.E.M. * *p* < 0.05, ** *p* < 0.01, *** *p* < 0.001; Scale bar = 100 µm; *n* = 16/group.

To understand the role of inflammasome formation, IHC, and Western blot analyses were performed in diabetic heart tissue +/− BMP7 treatment for TLR4-NLRP3 mediated cell death. A representative photomicrograph is shown for positive TLR4 (Figure 2A) and NLRP3 (Figure 2B) cardiomyocytes in the heart. Image quantification showed a significant increase of TLR4 (Figure 2C, *p* < 0.001) and NLRP3 (Figure 2E, *p* < 0.001) expression levels in cardiac myocytes in the STZ-induced diabetic cardiomyopathy group compared to the control. BMP-7 treatment against inflammasome formation showed a significant reduction (*p* < 0.001) in the expression of TLR4 and NLRP3 as compared to the diabetic cardiomyopathy group. To strengthen and confirm our IHC data, TLR4 and NLRP3 expression levels were determined by Western blot analysis and standardized using β-actin controls shown in Figure 2D,F, respectively. Densitometric analysis indicates significant increased expression of TLR4 (Figure 2D, *p* = 0.033) and NLRP3 (Figure 2F, *p* = 0.04) in the STZ group versus the control. However, treatment with BMP-7 attenuates the expression of TLR4 (Figure 2D, *p* = 0.027) and NLRP3 (Figure 2F, *p* = 0.033) compared to the STZ-induced diabetic cardiomyopathy. These results indicate the therapeutic effect of BMP-7 in the attenuation of TLR4 and NLRP3 inflammasome formation.

### 3.3. BMP-7 Inhibits Inflammasome Formation Protein Activators in Diabetic Hearts

To further understand the formation of protein activator Nek7-GBP5 in the inflammasome complex, which plays a role in the activation of specific complex protein NLRP3 [14,21], we performed Western blot analysis for Nek7 and GBP5 in the diabetic heart +/- BMP7 treatment. Our densitometric analysis shows an increased expression of Nek7 (Figure 2G, *p* = 0.007) and GBP5 (Figure 2H, *p* = 0.047) in STZ group versus control. Interestingly, treatment with BMP-7 attenuated the increased expression of Nek7 (Figure 2G, *p* = 0.038) and GBP5 (Figure 2H, *p* = 0.01) compared to the diabetic group, suggesting that BMP-7 attenuates inflammasome protein regulators Nek7 and GBP5 prior to the initiation of NLRP3 upregulation in diabetic cardiomyopathy.

### 3.4. BMP-7 Inhibits Pyroptotic Protein Caspase-1

**Figure 3:**
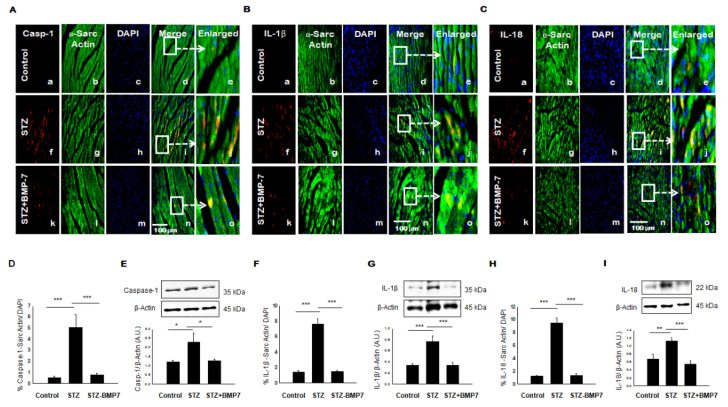
BMP-7 attenuates the expression of pyroptotic proteins caspase-1, IL-1β, and IL-18 in the diabetic heart. (**A**–**C**) Representative photomicrographs of heart sections stained in control group (a–e), STZ group (f–j) and STZ-BMP7 group (k–o) with sarcomeric-α actin (green, stained cardiac myocyte) (b, g, l) and pyroptotic markers caspase-1 (**A**), IL-1β (**B**) and IL-18 (**C**) in red (a, f, k) with nuclei stained (c, h, m) by DAPI in blue, merged (d, i, n) and enlarged (e, j, o) image. (**D**,**F**,**H**) Quantitative analysis-derived histograms of pryoptotic caspase-1 (**D**), IL-1β (**F**), and IL-18 (**H**) +ve cardiomyocytes (colocalization of both red and blue DAPI in green cardiomyocyte) were quantified over total DAPI in heart sections of mice. (**E**,**G**,**I**) Representative Western blots and densitometric analysis of caspase-1 (**E**), IL-1β (**G**), and IL-18 (**I**) markers are shown. Quantities are presented as an arbitrary unit (A.U). Statistical analysis was performed using One-Way ANOVA, which was followed by the Tukey test. Error bars= mean ± S.E.M. * *p* < 0.05, ** *p* < 0.01, *** *p* < 0.001; Scale bar = 100 µm; *n* = 16/group.

To clarify the effect of inflammasome formation in the induction of pyroptosis, we assessed the major pyroptotic protein caspase-1. A representative photomicrograph for heart IHC of caspase-1 is shown in Figure 3A. An increased expression of caspase-1 (Figure 3A(f)) was observed compared with the controls. Co-stained caspase-1 protein expression with sarcomeric α-cardiac myosin showed an increased expression of this caspase-1 present in the cardiac myocytes (Figure 3A(i)) and enlarged image (Figure 3A(j)). Our quantitative data showed a significant increase in caspase-1 (Figure 3D, *p* < 0.001) expression levels in the STZ-treated group compared to the control. BMP-7 treatment showed protective effects against pyroptotic protein expression compared to the diabetic cardiomyopathy, as indicated by a significant reduction (*p* < 0.001) in caspase-1 levels. In addition, our Western blot data corroborated our IHC findings. Protein expression of caspase-1 (Figure 3E, *p* = 0.01) was significantly increased in STZ group compared to the control. Importantly, BMP-7 therapy significantly reduced caspase-1 (Figure 3E, *p* = 0.019) compared to diabetic mice. All in all, BMP-7 reduced caspase-1, which represents the main pivotal target in the pyroptosis process.

### 3.5. BMP-7 Inhibits Pyroptotic Proteins IL-1β and IL-18

We further examined downstream to caspase-1 specific pyroptosis associated inflammatory cytokines IL-1β and IL-18, which was reported to play a role in pyroptosis [8]. Representative photomicrographs for heart IHC of IL-1β and IL-18 are shown in Figure 3B,C, respectively. We observed an increased expression of IL-1β (Figure 3B(f)) and IL-18 (Figure 3C(f)) in the diabetic heart compared with controls. Moreover, an increased expression of IL-1β (Figure 3B(i) and enlarged image, Figure 3B(j)) and IL-18 (Figure 3C(i) and enlarged image Figure 3C(j)) in cardiac myocytes when heart sections were co-stained with cardiac-specific α-SM actin. Quantitative data showed a significant increase of IL-1β (Figure 3F, *p* < 0.001) and IL-18 (Figure 3H, *p* < 0.001) expression levels in mice treated with STZ compared to control. BMP-7 treatment showed a significant reduction (*p* < 0.001) in the expression of IL-1β, and IL-18 compared to the diabetic cardiomyopathy. In addition, our Western blot data confirmed our IHC findings, as we observed a significant increase in protein expression of IL-1β (Figure 3G, *p* < 0.001) and IL-18 (Figure 3I, *p* = 0.008) in STZ group compared to the control. Importantly, BMP-7 treatment significantly reduced IL-1β (Figure 3G, *p* < 0.001) and IL-18 (Figure 3I, *p* < 0.001) compared to diabetic mice. Taken together, BMP-7 reduced the pyroptotic cascade IL-1β and IL-18 in the diabetic heart via caspase-1 dependent pathway. This suggests the potential for BMP-7 to reduce pyroptotic cell death in diabetic hearts.

### 3.6. BMP-7 Inhibits Pro-Inflammatory Cytokine IL-6 and TNF-α level in Diabetic Hearts

**Figure 4:**
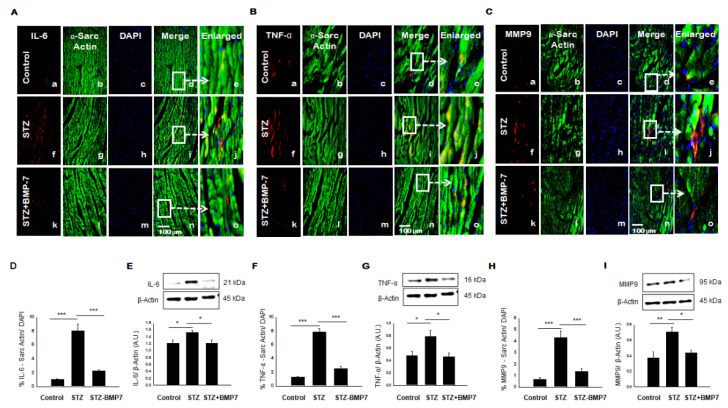
BMP-7 decreases the expression of inflammatory cytokines IL-6 and TNF-α, and proteolytic MMP9 in the diabetic heart. (**A**–**C**) Representative photomicrographs of heart sections stained in the control group (a–e), STZ group (f–j), and STZ-BMP7 group (k–o) with sarcomeric-α actin (green, stained cardiac myocyte) (b, g, l) and inflammatory markers IL-6 (**A**), TNF-α (**B**), and MMP9 (**C**) in red (a, f, k) with nuclei stained (c, h, m) by DAPI in blue, merged (d, i, n) and enlarged (e, j, o) image. (**D**,**F**,**H**) Quantitative analysis-derived histograms of IL-6 (**D**), TNF-α (**F**), and MMP9 (**H**) +ve cardiomyocytes (colocalization of both red and blue DAPI in green cardiomyocytes) were quantified over total DAPI in heart sections of mice. (**E**,**G**,**I**) Representative Western blots and densitometric analysis of IL-6 (**E**), TNF-α (**G**), and MMP9 (**I**) markers are shown. Quantities are presented as an arbitrary unit (A.U). Statistical analysis was performed using One-Way ANOVA, which was followed by the Tukey test. Error bars= mean ± S.E.M. * *p* < 0.05, ** *p* < 0.01, *** *p* < 0.001; Scale bar = 100 µm; *n* = 16/group.

During inflammation, IL-6 and TNF-α are known to be one of the most important pro-inflammatory cytokines [8]. Therefore, to determine the level of inflammation in the diabetic heart, IHC staining for IL-6 and TNF-α was performed. Representative pictures of IL-6 (Figure 4A) and TNF-α (Figure 4B) are shown in diabetic heart tissue +/− BMP-7 treatment. A statistically significant increased expression of IL-6 (Figure 4A(f)) and TNF-α (Figure 4B(f)) in the diabetic heart compared with controls and STZ-BMP7 groups. Quantitative data showed a significant increase of IL-6 expression (Figure 4D, *p* < 0.001) and TNF-α (Figure 4F, *p* < 0.001) in STZ diabetic mice compared to the control. BMP-7 therapy reduced significantly (*p* < 0.001) the expression levels of IL-6 and TNF-α compared to the diabetic mice. The results of the IHC data matched the results of the Western blot data, where protein expression of IL-6 (Figure 4E, *p* = 0.037) and TNF-α (Figure 4G, *p* = 0.024) was significantly increased in STZ diabetic mice compared to control. Moreover, exogenous BMP-7 attenuated the expression of IL-6 (Figure 4E, *p* = 0.039) and TNF-α (Figure 4G, *p* = 0.016) compared to the STZ diabetic mice, indicating that BMP-7 has an anti-inflammatory effect.

### 3.7. BMP-7 Inhibits MMP9 Level in Diabetic Hearts

MMP9 is a known matrix metalloproteinase (MMPs) released during inflammation and is heavily involved in cardiac fibrosis in diabetes [22]. Representative pictures of MMP9 in Figure 4C are shown in diabetic heart tissue +/− BMP-7 treatment. Our data suggested a significant increase in expression of MMP9 (Figure 4C(f,j)) in the diabetic heart compared with other groups. Our quantitative results demonstrated in STZ-induced diabetic mice a significant increase in MMP9 (Figure 4H, *p* < 0.001) relative to control. However, treatment with BMP-7 significantly diminished the expression levels of MMP9 (*p* < 0.001) compared to the diabetic mice. Parallel to our IHC data, we performed Western blot, which confirmed our IHC findings. Densitometric analysis showed that protein expression of MMP9 (Figure 4I, *p* = 0.004) was significantly increased in diabetic mice compared to control. Interestingly, treatment with exogenous BMP-7 reduced MMP9 expression (Figure 4I, *p* = 0.016) vs. STZ-administered mice. As such, these findings indicate that BMP-7 may play a role in improving myocardial remodeling.

### 3.8. BMP-7 Reduces Monocytes/Macrophages +ve Markers in Diabetic Hearts

**Figure 5:**
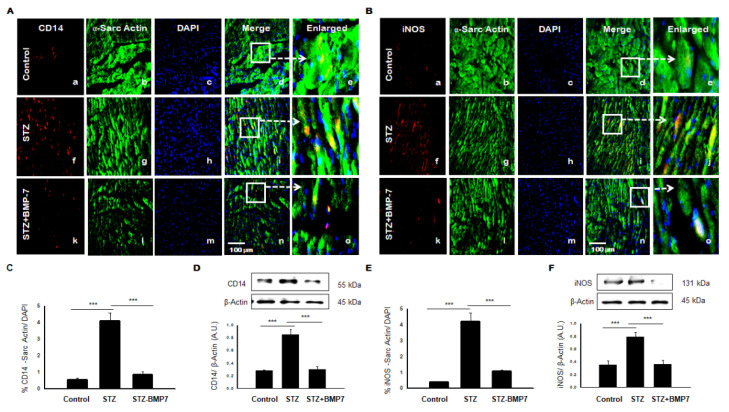
BMP-7 decreases expression of infiltrated monocyte (CD14) and macrophage M1 (iNOS) markers in the diabetic heart. (**A**,**B**) Representative photomicrographs of heart sections stained in the control group (a–e), STZ group (f–j) and STZ-BMP7 group (k–o) with sarcomeric-α actin (green, stained cardiac myocyte) (b, g, l) and CD14 (**A**) and iNOS (**B**) in red (a, f, k) with nuclei stained (c, h, m) by DAPI in blue, merged (d, i, n) and enlarged (e, j, o) image. (**C**,**E**) Quantitative analysis-derived histograms of CD14 (**C**) and iNOS (**E**) +ve cardiomyocytes (colocalization of both red and blue DAPI in green cardiomyocyte) quantified over total DAPI in heart sections of mice. (**D**,**F**) Representative Western blots and densitometric analysis of inflammasome CD14 (**D**) and iNOS (**F**) markers are shown. Quantities are presented as an arbitrary unit (A.U). Statistical analysis was performed using One-Way ANOVA, which was followed by the Tukey test. Error bars= mean ± S.E.M. *** *p* < 0.001; Scale bar = 100 µm; *n* = 16/group.

We assessed the source of pro-inflammatory cytokines, therefore, we assessed monocytes and macrophages using IHC analysis in the diabetic heart. Representative pictures of CD14, a marker for monocytes (Figure 5A), and iNOS, a marker for M1 macrophages (Figure 5B) are shown in the diabetic heart +/− BMP-7 treatment. Expression of CD14 (Figure 4A(f)) and iNOS (Figure 5B(f)) in the diabetic heart was an apparent increased compared with controls and STZ-BMP7 groups. Quantitative data showed a significant increase of CD14 expression (Figure 5C, *p* < 0.001) and iNOS (Figure 5E, *p* < 0.001) in STZ diabetic mice compared to the control. Treatment with BMP-7 decreased expression of CD14 and iNOS significantly (*p* < 0.001) compared to the diabetic groups. In addition, our Western blot data confirmed our IHC findings, where protein expression of CD14 (Figure 5D, *p* < 0.001) and iNOS (Figure 5F, *p* < 0.001) were significantly increased in STZ group compared to the control. Importantly, BMP-7 treatment significantly reduced CD14 (Figure 5D, *p* < 0.001) and iNOS (Figure 5F, *p* < 0.001) compared to diabetic mice. Taken together, BMP-7 reduced the infiltrated inflammatory monocytes and then polarized M1 macrophages (CD14/iNOS) in the diabetic heart.

### 3.9. BMP-7 Reduces Dendritic Cells (DCs) +ve Markers in Diabetic Hearts

**Figure 6:**
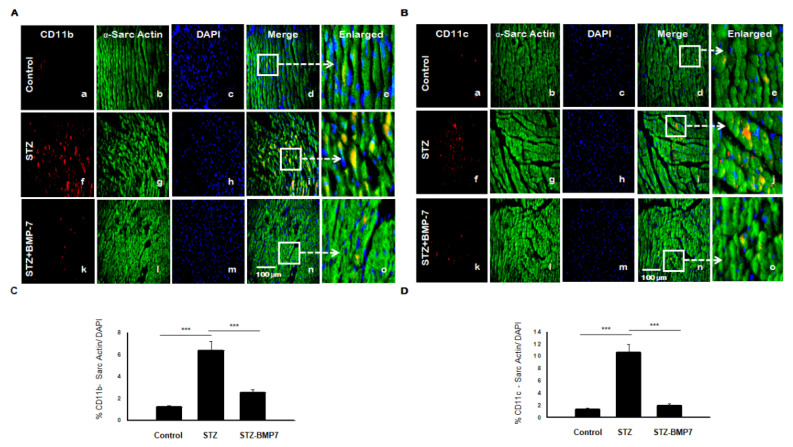
BMP-7 decreases the expression of inflammatory dendritic cells (CD11b and CD11c) in the diabetic cardiac heart. (**A**,**B**) Representative photomicrographs of heart sections stained in the control group (a–e), STZ group (f–j), and STZ-BMP7 group (k–o) with sarcomeric-α actin (green, stained cardiac myocyte) (b, g, l) and CD11b (**A**) and CD11c (**B**) in red (a, f, k) with nuclei stained (c, h, m) by DAPI in blue, merged (d, i, n) and enlarged (e, j, o) image. (**C**,**D**) Quantitative analysis-derived histograms of CD11b (**C**) and CD11c (**D**) +ve cardiomyocytes (colocalization of both red and blue DAPI in green cardiomyocyte) were quantified over total DAPI in heart sections of mice. Statistical analysis was performed using One-Way ANOVA, which was followed by the Tukey test. Error bars= mean ± S.E.M., *** *p* < 0.001; Scale bar = 100 µm; *n* = 16/group.

We assessed DCs markers CD11b and CD11c using IHC analysis in diabetic heart +/− BMP-7 treatment. Representative pictures of CD11b (Figure 6A) and CD11c (Figure 6B) are shown in the diabetic heart tissue. We observed an apparent increased expression of CD11b (Figure 6A(f)) and CD11c (Figure 6B(f)) in the diabetic heart compared with controls. Quantitative data showed a significant increase of CD11b expression (Figure 6C, *p* < 0.001) and CD11c (Figure 6D, *p* < 0.001) in STZ diabetic mice compared to control. In treatment with BMP-7, the expression of CD11b and CD11c were significantly (*p* < 0.001) reduced compared to the diabetic groups. Taken together, BMP-7 reduced the infiltrated inflammatory DCs in the diabetic heart.

### 3.10. BMP-7 Enhances the Recruitment of EPCs and Induces Neovascularization in Diabetic Hearts

**Figure 7:**
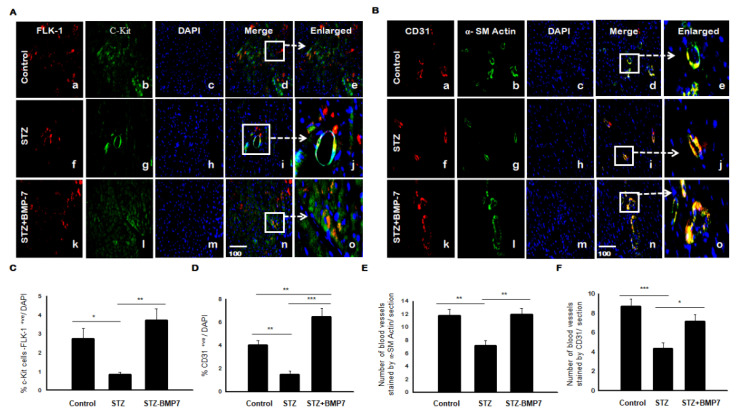
BMP-7 enhances the recruitment of c-kit, Flk-1, and CD31 cells with neovascularization in diabetic cardiac tissues. (**A**,**B**) Representative photomicrographs of heart sections stained in the control group (a–e), STZ group (f–j), and STZ-BMP7 group (k–o) with c-Kit (**A**) and α-SM actin (**B**) in green (b, g, l) and FLK-1 (**A**) and CD31 (**B**) in red (a, f, k) with nuclei stained (c, h, m) by DAPI in blue, merged (d, i, n) and enlarged (e, j, o) image. (**C**,**D**) Quantitative analysis of c-Kit/ Flk-1 (**C**) and CD31 (**D**) +ve cells (colocalization of both red and blue DAPI in cardiomyocyte) quantified over total DAPI in heart sections of mice. (**E**,**F**) The number of blood vessels stained with α-SM actin (green, **E**) and endothelial lining blood vessels CD31 (red, **F**) are counted in heart sections of mice. Statistical analysis was performed using One-Way ANOVA, which was followed by the Tukey test. Error bars = mean ± S.E.M. * *p* < 0.05, ** *p* < 0.01, *** *p* < 0.001; Scale bar = 100 µm; *n* = 16/group.

We further need to assess diabetic effects on blood vessel formation with and without BMP-7; therefore, we examined c-Kit/Flk-1 and CD31/α-SM actin using IHC analysis in the diabetic heart. Representative pictures of c-kit/Flk-1 and CD31/α-SM actin is shown in Figure 7A,B(d,i,n,e,j,o), respectively, in diabetic heart tissue +/− BMP-7 treatment. A statistically significant decreased expression of c-kit/Flk-1 (Figure 7A(f)) and CD31 (red expression in Figure 7B(f)) was shown in the diabetic heart compared with controls and STZ-BMP7 groups. Quantitative data showed a significant decrease of c-kit/Flk-1 expression (Figure 7C, *p* = 0.047) and CD31 (Figure 7D, *p* = 0.005) in STZ diabetic mice compared to the control. After treatment with BMP-7, expression of c-kit/Flk-1 (Figure 7C, *p* = 0.002) and CD31 (Figure 7D, *p* < 0.001) was increased significantly compared to the diabetic groups. In addition, CD31 was significantly increased in STZ-BMP7 group (Figure 7D, *p* = 0.007) versus control. Neovascularization data showed a significant decrease in the number of blood vessels stained by α-SM actin in the media of blood vessels (Figure 7E, *p* = 0.003) and the endothelial lining of blood vessel intima CD31 (Figure 7F, *p* < 0.001) in STZ diabetic mice compared to control. Following treatment with BMP-7, the number of blood vessels stained with α-SM actin (Figure 7E, *p* = 0.002) and CD31 (Figure 7F, *p* = 0.015) was increased significantly compared to the diabetic groups. These results confirmed that BMP-7 increased recruitment and homing of EPCs in diabetic hearts and enhanced neovascularization formation.

### 3.11. BMP-7 Decreases Inflammatory Cells and Hypertrophy in Diabetic Hearts

**Figure 8:**
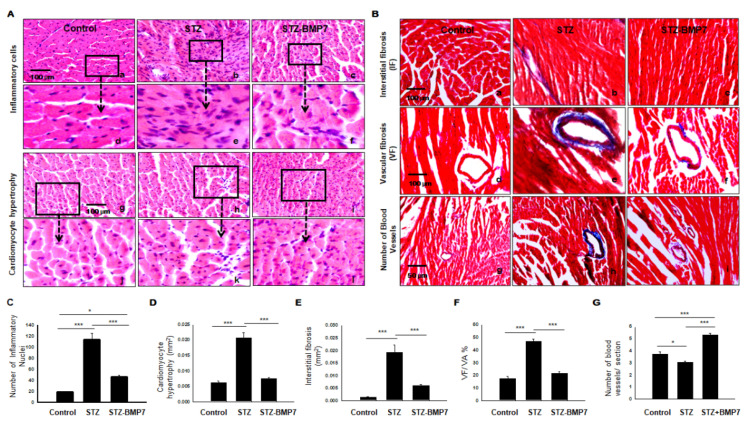
BMP-7 reduced cardiac infiltrated inflammatory cells, hypertrophy, interstitial, and vascular fibrosis in the diabetic heart. (**A**) Hematoxylin and Eosin staining (H&E) of heart sections showing inflammatory cells in images (a–c) and enlarged (d–f); hypertrophy in images (g–i) and enlarged (j–l) in all 3 groups. (**B**) Representative photomicrographs of Masson’s trichrome staining performed on heart sections to assess interstitial (a–c), vascular (d–f) fibrosis, and the number of blood vessels (g–i) in all 3 groups. (**C**–**G**) Quantitative analysis of the number of inflammatory cells (**C**) and cardiomyocyte cell diameter area (hypertrophy) (**D**), interstitial fibrosis (**E**), vascular fibrosis (**F**), and the number of blood vessels (**G**) in all groups. Statistical analysis was performed using One-Way ANOVA, which was followed by the Tukey test. Images taken at 40×; Error bars = mean ± S.E.M. * *p* < 0.05, *** *p* < 0.001; Scale bar = 100 µm; *n* = 16/group.

STZ-induced diabetic cardiomyopathy is known to increase the number of inflammatory cells and cardiomyocyte area (hypertrophy) in heart tissue [4]. H&E staining was performed on the transverse heart section and represented in Figure 8A. STZ hearts (b, h) showed infiltration of inflammatory cells, the architecture of cardiomyocyte hypertrophy, deformity in size and shape of nuclei, degenerated cytoplasm, and myofibrillar disarray versus controls (a, g). Controls demonstrated an oval, single shape for nuclei and were observed at the center of the cardiomyocytes. Quantitative analysis of infiltrated inflammatory cells (Figure 8C) showed higher numbers in the diabetic group (average number 113.3 ± 11.91) compared with those in the control group (average number 18.6 ± 1.02, *p* < 0.001) but were alleviated by BMP-7 treatment (average number 46.51 ± 2.91). Quantitative analysis of cardiomyocyte cell diameter area (Figure 8D) showed higher cardiomyocyte width in the diabetic group (0.021 ± 0.001 mm^2^) compared with those in the control group (0.006 ± 0.00 mm^2^, *p* < 0.001) but were reduced by BMP-7 treatment (0.007 ± 0.00 mm^2^). These results indicate that BMP-7 treatment attenuates cardiac inflammatory cells infiltrations and hypertrophy in diabetic cardiomyopathy.

### 3.12. BMP-7 Decreases Interstitial and Vascular Fibrosis in Diabetic Hearts

To measure the influence of BMP-7 on cardiac fibrosis in the diabetic heart, we determined collagen deposition using histological Masson’s trichrome (Figure 8B). Our representative photomicrographs showed interstitial fibrosis (a–c), vascular fibrosis (d–f), and the number of blood vessels (g–i) from all three groups. Quantitative analysis of the cardiac tissue was performed by direct measurement of the blue area using ImageJ software. Our data showed interstitial fibrosis was dramatically increased in the diabetic group (0.019 ± 0.003 mm^2^) relative to the control group (0.0014 ± 0.0001 mm^2^, *p* = 0.001; Figure 8E). However, after BMP-7 treatment, interstitial fibrosis was significantly decreased (0.005 ± 0.0004 mm^2^, *p* < 0.001) relative to the diabetic group (Figure 8E). Our vascular fibrosis in the diabetic group was significantly different compared to the control group (*p* < 0.001; Figure 8F), whereas the addition of BMP-7 significantly abrogated vascular collagen deposition in diabetic mice (*p* < 0.001). The number of blood vessels in the diabetic group was significantly lower relative to the control group (*p* = 0.014; Figure 8G), whereas the addition of BMP-7 was significantly associated with an increased number of blood vessels relative to diabetic mice (*p* < 0.001) and control (*p* < 0.001). Collectively, our data suggest that treatment with BMP-7 acts as anti-fibrotic as it minimizes and inhibits fibrosis formation in the diabetic heart. In addition, BMP7 enhances neovascularization marked by Masson’s staining in parallel to α-SM actin and CD31 immunostaining of blood vessels.

### 3.13. BMP-7 Improves LV Heart Function in Diabetic Mice

**Figure 9:**
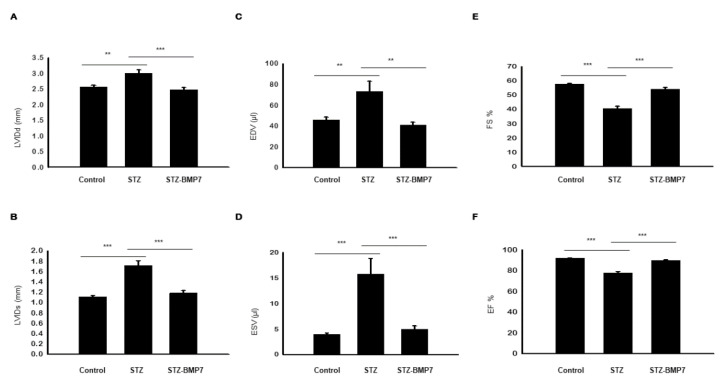
BMP-7 improves overall cardiac function in the diabetic heart. Representative histograms of transthoracic echocardiography data showing the mean ± SEM values for the control, STZ, and STZ-BMP-7 groups: (**A**) left ventricular (LV) internal dimension at end-diastole (LVIDd). (**B**) LV internal dimension at end-systole (LVIDs). (**C**) End-diastolic volume (EDV). (**D**) End-systolic volume (ESV). (**E**) Fractional shortening (FS%). (**F**) Ejection fraction (EF%). Statistical analysis was performed using One-Way ANOVA, which was followed by Tukey test; Error bars = mean ± S.E.M. ** *p* < 0.01, *** *p* < 0.001; *n* = 16/group.

At six weeks, our echocardiogram data shown in Figure 9A–F suggests impaired LV function in the STZ diabetic group as demonstrated by increased LVIDd (Figure 9A, *p* = 0.003), LVIDs (Figure 9B, *p* < 0.001), EDV (Figure 9C, *p* = 0.007), and ESV (Figure 9D, *p* < 0.001) versus the control group. Decreased LV-FS% (Figure 9E, *p* < 0.001) and LV-EF% (Figure 9F, *p* < 0.001) were observed for STZ treated mice compared with the control. A significant improvement in LV function was achieved after BMP-7 administration as indicated by decreasing LV remodeling parameters LVIDd (Figure 9A, *p* < 0.001), LVIDs (Figure 9B, *p* < 0.001), EDV (Figure 9C, *p* = 0.002), and ESV (Figure 9D, *p* < 0.001); and increased LV function parameters FS% (Figure 9E, *p* < 0.001) and EF% (Figure 9F, *p* < 0.001) relative to the diabetic group. All echocardiographic data, taken together, suggest that BMP-7 preserves diabetic cardiac systolic and diastolic dysfunction.

## 4. Discussion

Diabetic cardiomyopathy involves multiple pathophysiological changes such as downregulation of glucose transporter type-4 (GLUT4) recruitment, decrease in nitric oxide, increased reactive oxidative stress (ROS), collagen deposition/fibrosis, cell deaths (apoptosis, necrosis, and autophagy), and inflammation [2,3]. The impact of inflammation and inflammatory infiltrated cells on the microenvironment of a diabetic heart is complex and unexplained [23,24,25,26]. The present study using a type 1 diabetic model [27,28] focused on the types of inflammatory cell infiltration, inflammation associated with pyroptotic cell death, and the novel pyroptosis mechanisms involved with diabetic cardiomyopathy. Further, we discuss the potential therapeutic options with BMP-7.

Recent studies report that pyroptosis is present in many heart diseases such as Doxorubicin-induced cardiomyopathy [9], ischemia reperfusion injury [13], and diabetic cardiomyopathy [5,6]. Pyroptosis is activated through the binding of DAMPs or pathogen-associated molecular patterns (PAMPs), released by dead or dying cells in response to inflammation or infection with various types of bacteria and viruses [13,29]. Released DAMPs can bind directly to TLR4 and induce mitochondrial ROS, which can cause the activation of the NLRP3 inflammasome [7,8,11]. However, the initiation and regulation of pyroptosis involving the NLRP3 inflammasome complex, which activates caspase-1 in a diabetic heart, is not well established [11,12]. Further, the role of Nek7 and GBP5 in activation or regulation of NLRP3 inflammasome-mediated pyroptosis is being recognized in other diseases [12,14,21]. However, these cellular mechanisms have not been investigated in diabetic cardiomyopathy, which pose as a potential therapeutic target. As such, this serves as one of the novels focuses for this study as a possible mechanism that activates pyroptosis in diabetic cardiomyopathy.

Our results showed that the pyroptotic signaling cascade in diabetic cardiac tissue was prevalent due to the high expression of pyroptotic initiator TLR4, which formed the NLRP3 inflammasome complex, further activating the caspase-1-dependent pathway. These results were consistent with other studies showing higher expression of TLR4, NLRP3, and caspase-1 leading to pyroptosis of cardiomyocytes after induction with DAMPs in doxorubicin-induced toxicity studies [7,8,9,11]. Mechanistically, our results showed a significant increased expression of Nek7 and GBP5 in a diabetic heart, which was in congruence with other non-diabetic studies, suggesting the involvement of the Nek7-GBP5 pathway in the diabetic heart [12,13,14,21].

Subsequently, active caspase-1 promotes pro-IL-1β and pro-IL-18 maturation and cleaves gasdermin-D proteins, which induce an inflammatory reaction to further perpetuate pyroptosis in the cardiomyocyte [7,11,13]. Our results showed that diabetic cardiac tissue was associated with a high-inflammatory reaction due to the significant presence of IL-1β and IL-18, suggesting the presence of pyroptosis in the diabetic cardiomyocytes. Furthermore, we examined the levels of inflammatory cytokines IL-6 and TNF-α, which were significantly upregulated, implying that inflammation is prevalent in diabetic heart tissues, which raises further concerns on the source of inflammatory cytokines and their impact on the heart.

Increased levels of peripheral leukocytes, inflammatory monocytes and mature DCs, and other inflammatory cells are present in the diabetic blood [25,30]; however, whether they infiltrate into diabetic heart tissue is a current subject of the investigation that seems to be much more complex than previously thought. Thus, we explore the presence of pro-inflammatory infiltrated cells in the diabetic heart.

The current study showed higher expression of mature monocyte markers, CD14 and CD11b, in diabetic heart tissue. These results were consistent with other non-diabetic studies such as myocardial infarction and myocarditis [23,25,30]. Further, the current study showed higher expression of mature pro-inflammatory M1 macrophage markers, iNOS and CD11b, in diabetic heart tissue. In addition, this study showed higher expression of mature DCs markers, CD11b, and CD11c, in diabetic hearts. These results were consistent with other heart studies [24,26,31,32].

Diabetes is being associated with neutrophil activation and release of neutrophils extracellular traps (NETs), which induce sustained inflammation and has a role in activation as well as recruitment of monocytes and macrophages in diabetic cardiomyopathy [23,25,33]. Neutrophils (N1 phenotype) induce pro-inflammatory properties in the early stage and can polarize to anti-inflammatory (N2 phenotype) in the late and healing stage [25]. Hence, our results showed that inflammatory infiltrated cells and cardiomyocyte hypertrophy were increased in H&E-stained diabetic heart sections compared to the control. Therefore, we anticipate neutrophils to migrate to the site of inflammation in diabetic patients, secrete cytokines and growth factors IL-8, IL-1β, TNF-α, and IL-1ra, proteolytic (MMP9 and MMP12) and enhance oxidative stress and apoptosis [25,33].

Further, cardiac remodeling biomarkers MMP9 expression (zinc-dependent collagenase) was increased and impaired myocardial contraction in diabetic cardiomyopathy [22,34]. In addition, MMP9 tissue inhibitor metalloproteinase (TIMP-1) ratio was increased 3–5 times in patients with dilated cardiomyopathy [34]. Our results showed cardiac interstitial and vascular fibrosis with increased expression of MMP9 in diabetic heart tissues [4,35,36,37]. Our data showed a significant increase in MMP9 levels, which is released by infiltrated inflammatory monocytes or M1 macrophages as published previously [23]. Our data are consistent with other published studies that showed infiltrated monocytes upregulating MMP9 levels [22,23,34]. Furthermore, released MMP9 shows increased cardiac fibrosis associated with decreased cardiac function suggesting altered structural changes in the diabetic heart, which is in agreement with published studies [22,23,34].

The association of pyroptosis with EPCs has not been investigated in current diseased heart studies. This warranted understanding the exact cause of decreased EPCs population and neovascularization with respect to inflammation and pyroptosis in the diabetic heart. Therefore, we determined whether cardiac fibrosis and altered structural changes in the heart have effects on the number of EPCs and their potential to regenerate injured myocardium. Our data showed a lower expression of EPCs recruitment (c-Kit/FLk1 and CD31) in diabetic heart tissues and a lower level of neovascularization, suggesting that diabetic EPCs are defective, or they have become less proliferative due to fibrosis as well as inflammation and cell death in cardiomyocytes. Our data are in association with depletion of mobilization in the circulating EPCs as well as vascular endothelial dysfunction in atherosclerosis and myocardial remodeling [38,39]. Taken together, our results showed higher pyroptosis and inflammatory cytokines, infiltrated inflammatory cells markers, less vascularization, and finally EPCs dysfunction leading to adverse remodeling in the diabetic heart.

In a clinical setting, hypoglycemic drugs such as sodium-glucose cotransporter 2 inhibitors (SGLT2i) (Empagliflozin, Canaglifozin, Ipragliflozin and Dapagliflozin) [40], and glucagone-like peptide-1 (GLP-1) receptor analogs (Liraglutide, Semaglutide, Exenatide, and Albiglutide) [41], or other drugs such as IL-1β antibodies antagonist (Canakinumab or Gevokizumab) [42], and recombinant IL-1β receptor antagonist (Anakinra) [43] decrease cardiovascular events and mortality in patients with type-2 diabetes [1,3,41,43]. However, these treatments for diabetic cardiomyopathy are limited in use due to their cost and side effects, including dyslipidemia, neutropenia, infection, muscle aches and myalgia, poor solubility, and hepatoxicity [40,42,44,45]. Therefore, it is imperative to find alternative treatments for cardiomyopathy induced by diabetes. Recently, the Food and Drug Administration (FDA) approved rhBMP-7 (OP-1^®^ Putty, Stryker, Biotech) as an effective therapy in osteoporosis and repair of critical-size bone defect in nonunion bone fracture [17]. However, it is unknown whether BMP-7 treatment can attenuate pyroptosis and its signaling pathway that can be used as an adjuvant with hypoglycemic drugs in diabetic cardiomyopathy.

Notably, BMP-7 is a growth factor that binds directly with high affinity to membrane BMP receptor type I (activin-like kinase; ALK-1, 2, 3, 6), which requires the activation from BMP receptor type II kinase [7,35]. Once activated, a canonical (Smad dependent) signaling pathway induces Smads 1, 5, and 8 complex, which binds to another coactivator Smad 4 and enters to the nucleus to activate and regulate specific genes [36,37]. In addition, BMP-7 activates the non-canonical (Smad independent) signaling pathway through mitogen-activated protein kinase (MAPK), extracellular signal-regulated kinase (ERK), Jun N-terminal kinase (JNK), and p38 mitogen–activated protein kinase (P^38^) to activate target genes [35,37].

Our results showed that inflammasome proteins (TLR4 and NLRP3), NLRP3 activators (Nek7 and GBP5), and inflammatory cytokines (IL-1β, IL-18, IL-6, and TNF-α) were significantly reduced with the administration of BMP-7 in diabetic mice, suggesting BMP-7 as a potent anti-inflammatory and anti-pyroptotic agent. Next, inhibition of Nek7 and GBP5 that disrupts the NLRP3 inflammasome formation and ultimately pyroptosis with BMP-7 treatment is interesting and suggests pathway-specific inhibition of pyroptosis in the diabetic heart.

Our results of BMP-7 are consistent with other specific inhibitors to attenuate pyroptosis such as TLR4 inhibitors (TAK-242 and Eritoran) [46], NLRP3 inhibitors (MCC950, β-Hydroxybutyrate, CY-09, OLT1177) [44], Nek7- NLRP3 polymerization inhibitor (Oridonin) [47], caspase-1 inhibitor (VX-765, VX-740 as Pralnacasan) [48], IL-1β inhibitor [42,43], TNF-α inhibitor [49] and IL-6 inhibitor [45].

Next, results showed that inflammatory infiltrated cells (monocytes-CD14, macrophages-iNOS, dendritic cells-CD11b, and CD11c) were significantly reduced with the administration of BMP-7 in diabetic mice, suggesting BMP-7 inhibits inflammatory monocyte/macrophages (M1) and mature DCs. Our current study results are consistent with results of BMP-7 that reduced inflammatory cytokines and differentiation of monocytes in our previously published studies of pre-diabetic heart [4] and atherosclerotic mice [16]; and reduced inflammatory mucosal epithelium DCs in other studies [50].

Our results showed BMP-7 enhances EPCs and neovascularization in diabetic cardiomyopathy, this observation was in agreement with other unrelated studies using rhBMP-7 or stem cell overexpressing BMP7- in repairing the nonunion fracture [51,52], or with other types of BMP effects as BMP-9 to improve neovascularization of hind limb ischemia [53].

Our results of BMP-7 reduced adverse myocardial remodeling MMP9 marker, hypertrophy as well as interstitial and vascular fibrosis. There is only one study that showed BMP-7 reduced MMP9 in an inflammatory silica-induced lung cell line in vitro [54]. Therefore, it is possible that BMP-7 reduced cardiac fibrosis and adverse myocardial remodeling through transforming growth factor (TGF-β1) [4,35,36,37].

Finally, heart function was measured using echocardiography to examine the diabetes-induced cardiomyopathy and the therapeutic effect of BMP-7 in improving heart functionality. Our echocardiography data showed improved heart function after administration of BMP-7 compared to diabetic mice by increasing LV-EF% and FS%. These results corroborate our pre-diabetic cardiomyopathy [4], ischemic and hypertrophic cardiomyopathy [35,36,37]. Despite the fact that BMP-7 is not considered as an inotropic drug, BMP-7 has shown the capability of improving LV function due to reversing cardiac remodeling [4,35,36,37].

## 5. Conclusions

In conclusion, our previous pre-diabetic [4] and Type-1 diabetic muscle [7] studies suggested BMP-7 decreases hyperglycemia as shown in graphical abstract. Here, we report inflammation-induced cell death mediated by inflammatory cells (macrophage and dendritic cells) and inflammasome formation (TLR4, NLRP3) and NLRP3 activators (Nek7 and GBP5) that cause cardiac cell death is the key player in the development and progression of diabetic cardiomyopathy. BMP-7, a unique growth factor that we reported in this study, significantly reduces inflammation-induced cardiac cell death, reduces infiltrated macrophages and dendritic cells, decreases adverse cardiac remodeling, and enhances cardiac regeneration with improved cardiac function in pre-clinical studies of mice could have significant potential as a future therapy for diabetic patients.

## Data Availability

Data are contained within the article.
